# Clinical Features and Risk Factors of Fungal Peritonitis in Children on Peritoneal Dialysis

**DOI:** 10.3389/fped.2021.683992

**Published:** 2021-06-30

**Authors:** Xiaoyan Fang, Jingyi Cui, Yihui Zhai, Jiaojiao Liu, Jia Rao, Zhiqing Zhang, Jing Chen, Jialu Liu, Qianfan Miao, Qian Shen, Hong Xu

**Affiliations:** Department of Nephrology, Children's Hospital of Fudan University, National Children's Medical Center, Shanghai, China

**Keywords:** fungal peritonitis, peritoneal dialysis, risk factor, pathogen, children

## Abstract

**Objective:** To analyse the clinical manifestations, aetiology, prognosis, and risk factors of fungal peritonitis (FP) in children on peritoneal dialysis (PD).

**Methods:** Among 322 children undergoing PD at Children's Hospital of Fudan University, between January 2001 and December 2019, FP cases were retrospectively analysed and compared with those of bacterial peritonitis (BP) to analyse the risk factors of FP.

**Results:** A total of 124 cases of peritonitis were treated, including 11 FP cases in 11 children (0.0019 episodes/patient^*^month) and 113 BP cases in 64 children (0.02 episodes/patient^*^month). Among the 11 FP cases, 7 cases (63.64%) were caused by Candida and Candida parapsilosis (5/7) was the most common pathogen of Candida. All FP patients were converted to haemodialysis (HD) and did not resume PD during follow-up. Two patients (18.2%) died after 6 months of HD due to heart failure, 2 patients underwent kidney transplant after 2 years of infection, and the other 7 patients were still on HD. The univariate analysis showed the usage rate of antibiotics in the month before the onset of peritonitis was higher (45.45 vs. 15.93%) and the mean serum albumin was lower (31.4 vs. 34.4 g/L) in the FP group when compared with BP group (*P* < 0.05), while multivariate analysis showed that serum albumin ≤ 30 g/L was an independent risk factor for FP (odds ratio 4.896, 95% confidence interval 1.335–17.961).

**Conclusion:** FP is a rare complication of PD in children, but it is associated with high technique failure. Attention should be paid to hypoproteinaemia and antibiotic use in children on PD.

## Introduction

Peritoneal dialysis (PD) is an important renal replacement therapy for children with end-stage renal disease (ESRD). With advancements in PD connexion systems and dialysate biocompatibility, the incidence of PD-associated peritonitis is declining. However, PD-associated peritonitis is still a common serious complication of PD. It is the main cause of discontinuation of PD and death, and peritonitis itself is an independent risk factor for death. Dialysis-associated bacterial peritonitis (BP) in children is the most common type of peritonitis (accounting for ~80% of cases), while fungal peritonitis (FP) is less common, accounting for ~2–10.3% of all paediatric and adult cases (1–3). However, FP often leads to serious consequences with a mortality rate of 50%, and one of the main reasons is delayed diagnosis. FP is also the main cause of discontinuation of PD (4, 5). Few cases of PD-associated FP in children have been reported, especially in China. As a report of the Standardizing Care to Improve Outcomes in Paediatric End Stage Renal Disease (SCOPE) Collaborative in 2017, a large study on FP in children showed that most FP cases occurred in children under 2 years old and did not identify other risk factors (6).

This study retrospectively analysed children of peritonitis treated at Children's Hospital of Fudan University between January 2001 and December 2019 and conducted the analysis of FP cases that included clinical data, clinical manifestations, aetiology, prognosis, and risk factors. This study also compared the data with those of BP to improve the understanding of FP and provide a reference to help clinicians facilitate infection prevention, early detection, early treatment, and optimal prognosis.

## Methods

### Data Source and Study Population

Children with PD-associated peritonitis who received PD at Children's Hospital of Fudan University, between January 2001 and December 2019 were eligible for this study. Exclusion criteria were as follows: children with ESRD who received PD for <3 months.

For patients with clinical symptoms of peritonitis, a turbid dialysate sample was sent for laboratory tests, including WBC count and differential, smear, and culture (blood culture flask). Data collected consisted of the following: age at initial dialysis, sex, the primary aetiology of ESRD, peripheral blood haemoglobin, serum parathyroid hormone, serum albumin, routine peritoneal dialysate (WBC, neutrophils %), and G test (mycodextranase). Outcomes included kidney transplant, conversion to other dialysis modality, and 1-year mortality following infection. The study was approved by the ethical committee of Children's Hospital of Fudan University. Written informed consent was obtained from their patients or legal guardians.

### Definitions

According to the 2012 International Society for Peritoneal Dialysis (ISPD) guidelines (7), PD-associated peritonitis is diagnosed if 2 of 3 the following criteria are met: (1) clinical symptoms of peritonitis (abdominal pain, cloudy dialysate, fever); (2) peritoneal effluent leucocyte count > 100^*^ 10^6^/L and a differential of >50% polymorphonuclear cells; (3) positive dialysate culture.

Procedures for obtaining fungal cultures were as per the 2012 ISPD guidelines whereby for patients on APD without a day dwell, the fill volume should be instilled for a minimum of 1–2 h, with the subsequent effluent being sent for cell count, differential count, and culture. For patients on APD with a daytime exchange, after a dwell time of at least 2 h, then the effluent sent to test. The peritoneal effluent draw into two blood-culture bottles (7).

Fungal peritonitis was defined by the presence of a positive yeast culture and/or positive G test. To rule out a false-positive G test factors, such as the presence of intravenous blood products, contamination, and polymyxin must be ruled out.

Peritonitis rate was defined as the number of episodes of peritonitis divided by the total patient's months on PD. Prior antibiotic use was defined as use of antibiotic treatment, whether orally, intravenously or intraperitoneally, for peritonitis or for any other infection in the month prior to the fungal peritonitis.

### Statistical Analysis

Statistical analysis was performed using SPSS 25.0. The median interquartile range was used to describe the non-normally distributed measurement data, such as age, mean time from catheter placement to peritonitis, peritoneal effluent leucocyte count, serum albumin, peripheral blood haemoglobin, parathyroid hormone, hospital stay and healthcare expenses. Statistical analysis was performed by Wilcoxon rank-sum test. Frequency and percentage were used to describe categorical variables such as sex, primary kidney disease, prior antibiotic use and 1-year survival rate. The chi-square test was performed for intergroup analysis. Binary logistic regression was performed to analyse the risk factors for peritonitis. *P*-values < 0.05 were considered statistically significant.

## Results

### General Information

A total of 322 patients received PD at Children's Hospital of Fudan University, between January 2001 and December 2019. Of these, 124 had peritonitis, including 11 FP cases (8.87%) in 11 children (0.0019 episodes/patient^*^month) and 113 BP cases in 64 children (0.02 episodes/patient^*^month). At the time of FP diagnosis, the mean age was 8.5 years old (IQR 7.1–11.4). Six were boys and the other 5 were girls. The patients were on PD for an average of 2.3 years (IQR 1.3, 2.8) prior to FP. The average time from the onset of peritonitis to catheter removal was 0.4 months (IQR 0.2, 0.8). Clinical manifestations were turbid dialysate (11/11), abdominal pain (10/11), and fever (8/11). Five patients (45.45%) received antibiotics within 1 month before the FP onset, and 8 patients (72.73%) had a history of BP (Table 1).

**Table 1:** Clinical and microbiological characteristics of the FP group (*n* = 11).

**No**.	**Sex/age (years)**	**Primary disease**	**Time on PD (years)**	**Clinical symptoms**	**AB within 1 month before FP/route of antibiotic administration**	**History of fungal prophy**	**Fungus cultured from PD fluid fungal/G test**	**Medication**	**Time of catheter removal (months)**	**History of peritonitis**	**Prognosis**
1	M/15.5	Alport syndrome	5.5	Fever, abdominal pain	No		*Candida lusitaniae*/positive G test	Fluconazole	0.43	Once	HD
2	F/7.7	CNS	4.2	Fever, abdominal pain	No		*Candida parapsilosis*/positive G test	Fluconazole	0.27	Once	HD/transplant after 3 years
3	M/10.1	CAKUT	2.6	Fever, abdominal pain	No		*Candida parapsilosis*/positive G test	Fluconazole	0.20	Once	HD
4	M/11.0	NPH	0.6	Abdominal pain	No		Curvularia/positive G test	Fluconazole, micafungin, voriconazole	0.87	None	HD
5	F/11.8	Proteinuria	1.1	Fever, abdominal pain	No		*Candida parapsilosis*/positive G test	Fluconazole	0.07	None	HD/transplant after 2 years
6	M/7.4	CAKUT	2.3	None	Yes/IP	No	*Candida tropicalis*/positive G test	Itraconazole	0.33	Twice	HD/died after 6 months
7	F/2.6	Unknown	1.8	Abdominal pain	No		*Candida parapsilosis*/NA^*^	Fluconazole, voriconazole	1.07	None	HD/died after 6 months
8	M/14.9	Unknown	3.0	Fever, abdominal pain	Yes/IP	No	*Candida parapsilosis*/NA^*^	Micafungin, caspofungin, fluconazole	3.10	Once	HD
9	M/8.5	CAKUT	1.4	Fever, abdominal pain	Yes/IP	Yes	Positive G test	Fluconazole	0.67	Twice	HD
1 0	F/6.8	FSGS	2.5	Fever, abdominal pain	Yes/IV/IP	No	Positive G test	Fluconazole	0.10	Twice	HD
1 1	F/6.7	CAKUT	0.5	Fever, abdominal pain	Yes/IV/IP	No	Positive G test	Fluconazole	0.53	Twice	HD

*FP, fungal peritonitis; AB, antibiotics; M, male; F, female; CNS, congenital nephrotic syndrome; CAKUT, congenital anomalies of the kidney and urinary tract; NPH, Nephronophthisis; FSGS, focal segmental glomerulosclerosis; PD, Peritoneal dialysis; HD, haemodialysis; NA, not available; IV, intravenous; IP, intraperitoneal*.

* *Transferred from another hospital*.

Among the 11 FP cases, 7 (63.6%) were caused by Candida, such as *Candida parapsilosis* (*n* = 5), *Candida tropicalis* (*n* = 1) and *Candida lusitaniae* (*n* = 1). One case was caused by *Curvularia*, and the other 3 cases had negative cultures but were clinically diagnosed due to a positive G test, apparent peritoneal adhesions, and response to antifungal therapy. G tests were positive in 9 children, while the other 2 children who were transferred to our hospital, the G test was not performed. Detail antifungal therapy was listed in Table 1.

All patients were converted to haemodialysis (HD) and did not resume PD during follow-up. Two patients (18.2%) died after 6 months of HD due to heart failure, 2 patients underwent kidney transplant after 2 years of infection, and the other 7 patients were still on HD (Table 1). None of these patients in the FP sample had recent gastrostomy, ureterostomy and ostomy procedures before fungal peritonitis.

### Three Cases With Negative Culture and Positive G Test

3/11 of the FP cases had negative cultures but were clinically diagnosed with fungal infection due to positive G tests and responses to anti-fungal therapy. In case 9, the patient had BP 3 weeks before the onset of FP, with uncontrolled fungal infection, increasing dialysate WBC count, negative dialysate culture, and increasing G test readings (185.8–218.8-417 pg/ml). He was finally converted to long-term HD due to severe peritoneal adhesions. In case 10, the patient had BP for 1 month, with recurrent high cell counts in dialysate and increasing G test readings (121.8–359.4 pg/ml). Fungal infection was considered, the dialysis catheter was removed, and the patient was given fluconazole with good response and converted to HD. In case 11, the patient had BP for 1 month, with recurrent high cell counts in dialysate and increasing G test readings (728.1–1,000 pg/ml). Fungal infection was considered, the dialysis catheter was removed, and the patient was given fluconazole. Four weeks later, severe peritoneal adhesions were found during laparoscopic catheter placement, and the patient was converted to long-term HD.

### Comparison of Risk Factors and Prognosis Between FP and BP in Children

No significant intergroup differences were noted in age, sex, and the type of primary kidney disease between FP group and BP group. The univariate analysis showed the usage rate of antibiotics in the month before the onset of peritonitis was higher (45.45 vs. 15.93%) and the mean serum albumin was lower (31.4 vs. 34.4 g/L) in the FP group when compared with BP group (*P* < 0.05) (Table 2). In addition, multivariate analysis showed that serum albumin ≤ 30 g/L was the only independent risk factor for FP (odds ratio 4.896, 95% confidence interval 1.335–17.961). No significant intergroup differences were noted in peritoneal effluent leucocyte count, differential, haemoglobin and parathyroid hormone between 2 groups (Table 2). Moreover, hospital stays were longer (1.43 vs. 0.57 months), healthcare expenses were higher (80,480.19 vs. 21,390.97 RMB), the rate of conversion to HD was higher (100 vs. 5.3%), and 1-year survival rate after infection was lower (81.82 vs. 100%) in the FP group than those in the BP group (*P* < 0.05) (Table 3).

**Table 2:** Comparison of risk factors between FP and BP [median (P25, P75), *n* (%)].

	**FP (*n* = 11)**	**BP (*n* = 113)**	****χ**^**2**^/Z**	***P***	**Multivariate analysis**	
					**OR (95% CI)**	***P***
**Sex (*****n*****)**			0.000	1.000		
Male	6 (54.55)	62 (54.87)				
Female	5 (45.45)	51 (45.13)				
Age at the onset of peritonitis (years)	8.54 (6.9, 11.93)	10.91 (6.56, 13.95)	−0.549	0.590		
Diagnosis of primary disease (*n*)			0.720	0.982		
CNS/FSGS	2	24				
CAKUT	5	43				
Glomerulonephritis	2	16				
Cystic kidney disease	1	15				
Urinary calculi	0	1				
Unknown	1	14				
Mean time from catheter placement to peritonitis (years)	2.41 (1.13, 3.42)	1.19 (0.50–2.29)	−1.885	0.059	1.198 (0.845, 1.699)	0.311
Antibiotic within 1 month before FP (*n*)	5 (45.45)	18 (15.93)	5.780	0.0162	3.299 (0.815, 13.353)	0.094
Positive culture at the outlet (*n*)	4 (36.36)	25 (22.12)	1.130	0.286		
Dialysate WBC (10^6^/L)	1,145 (890, 4,521)	1,550 (480, 5,700)	−0.146	0.884		
Dialysate N (%)	71 (55, 85)	80 (70, 90)	−1.515	0.130		
**Albumin level (g/l)**						
Mean serum albumin	31.4 (23.1, 33.8)	34.4 (32.0, 38.9)	−2.421	0.015		
Serum albumin ≤ 30 (*n*)	5 (45.45)	16 (14.55)	4.68	0.035	4.679 (1.198, 18.271)	0.026
Serum albumin > 30 (*n*)	6 (54.55)	94 (85.45)				
Haemoglobin (g/L)	104 (83, 107)	105 (97, 122)	−0.510	0.610		
Parathyroid hormone (pmol/L)	14.70 (7.26, 65.79)	22.95 (8.53, 67.18)	−0.349	0.727		

*FP, fungal peritonitis; BP, bacterial peritonitis; CNS, congenital nephrotic syndrome; FSGS, focal segmental glomerulosclerosis; CAKUT, congenital anomalies of the kidney and urinary tract; N, Neutrophils*.

**Table 3:** Comparison of prognosis between FP and BP [median (P25, P75), *n* (%)].

	**FP**	**BP**	****χ**^**2**^/Z**	***P***
Hospital stay (months)	1.43 (0.83 1.90)	0.57 (0.47, 0.86)	−3.3842	<0.0001
Healthcare expenses (RMB)	80,480.19 (41,490.54, 204,635.15)	21,390.97 (16,482.94, 34,289.79)	−4.140	<0.0001
Conversion to HD	11 (100.00)	6 (5.30)	68.18	<0.0001
1-year survival rate after infection	9 (81.82)	113 (100.00)	^*^	0.007

*FP, fungal peritonitis; BP, bacterial peritonitis; HD, haemodialysis*.

* *Fisher test*.

## Discussion

In this study, a total of 124 cases of peritonitis occurred in 322 patients on PD at our hospital during 10-year interval, including 11 FP cases (8.87%) and 113 BP cases (92.12%). 7 of the 11 (63.6%) FP cases were caused by Candida. All 11 (100%) FP patients were converted to HD, and the 1-year survival rate was 81.82% after infection. Univariate analysis showed that mean serum albumin was lower (31.4 vs. 34.4 g/L) and the usage rate of antibiotics during the month before the onset of peritonitis was higher (45.45 vs. 15.93%) in the FP group than those in the BP group, while multivariate analysis showed that serum albumin ≤ 30 g/L was a major risk factor for FP.

Till now, PD-associated peritonitis remains a common and serious complication of PD. In particular, severe or recurrent peritonitis leads to structural and functional changes in the peritoneum and ultimately leads to peritoneal failure. It is a major cause of PD failure and conversion to long-term HD (6). Although FP is rare in children and adults on PD, it is associated with high mortality and technical failure rates. The 2012 ISPD peritonitis prevention and treatment guidelines state that dialysis centres should monitor the infection rate, pathogenic bacteria, and drug sensitivity of peritonitis to control the overall incidence of peritonitis at 0.5 cases per year or below (7).

FP may be caused by yeast-like fungi or filamentous fungi. Common yeast-like fungi include *Candida, Trichosporon*, and *Cryptococcus*. Filamentous fungi are rare in FP and include *Aspergillus, Rhizopus*, and *Histoplasma* (8). In this study, 7 (63.6%) cases were caused by *Candida* and *Candida parapsilosis* was the most common pathogen, accounting for 5 FP cases (45.5%), while other fungi are less common. For example, in case 4, *Curvularia* was identified only at the third culture attempt at 3 weeks after onset. *Curvularia* infection is rare, with a low positive rate. A previous study reported 7 cases of PD-associated *Curvularia* peritonitis. Of these, 6 patients had a history of peritonitis, 1 patient died, and the dialysis catheter was removed in all cases. For the death case, microscopy did not identify fungal hyphae until day 9 of treatment, and the death was related to the patient's advanced age (85 years) and lack of treatment response (9).

In this study, 3 of the FP cases had negative cultures but were clinically diagnosed with fungal infection due to positive G tests and responses to anti-fungal therapy. In all these 3 cases, dialysate culture was negative, and antibiotic therapy was infective. Therefore, FP was considered due to positive G tests. Clinical symptoms alleviated and G test results improved after catheter removal and fluconazole treatment. Clinically, fungal culture is the main test for fungal infections. However, a fungal culture may be affected by the conditions of the sampling, petri dishes, and culture equipment used. The symptoms of FP are similar to those of BP and include fever, abdominal pain, and turbid dialysate, which may lead to missed diagnoses in cases of negative fungal culture. Moreover, fungal culture is time-consuming, which may impede early diagnosis and treatment. New serological diagnostic assays, such as the G test and galactomannan (GM) test, are designed to detect deep fungal infections with a high detection rate (10, 11). The G test as a direct method to measure the (1/3)-b-D-glucan (BDG) which is a component of the cell wall of most fungi. BDG has been included in the relevant European Organization for Research and Treatment of Cancer/Mycoses Study Group (EORTC/MSG) diagnostic criteria for fungal infection (12). A meta-analysis of relevant studies about 2979 patients (594 with proven or probable invasive fungal infections), included in 16 studies, were showed that the pooled sensitivity of BDG was 76.8% [95% confidence interval (CI), 67.1–84.3%], and the specificity was 85.3% (95% CI, 79.6–89.7%). BDG has good diagnostic accuracy for distinguishing invasive fungal infections (13). However, the G test and GM test cannot identify the fungal species and genus, which may lead to misdiagnosis (false positives). Each assay has its own pros and cons, and assays may be combined to help diagnose deep fungal infections, especially when antibiotic therapy is ineffective (14). The results are meaningful if confounding factors of the G test and GM test (such as intravenous blood products, contamination, and polymyxin) are ruled out and 2 or more consecutive G tests and GM tests are positive (especially in the case of increasing readings) (15). In this study, 2 of the 11 FP cases were transferred from another hospital (cases 7, 8). The remaining 9 cases (81.82%) had positive G tests after the onset of FP. After catheter removal, additional G tests may be performed to evaluate the severity of fungal infection and guide the use of antifungal drugs. A study of adult patients showed that (1, 3)-β-D-glucan combined with galactomannan, which allowed to enhance the diagnosis rate of invasive fungal infections (16). Dialysate culture combined with the G test and GM test provides new possibilities for the early diagnosis of FP in order to prevent treatment delay and protect peritoneal function.

This study analysed the data of FP and BP cases and found that mean serum albumin was significantly lower in the FP group (31.4 vs. 34.4 g/L). Multivariate analysis showed that serum albumin ≤ 30 g/L was a major risk factor for FP. Previous study in Turkey reported that among 86 cases of peritonitis, 9 were FP (10.5%), and these had significantly lower serum albumin than the BP cases (17). Hypoproteinaemia is often reported as a risk factor for FP; it usually indicates malnutrition and chronic inflammation in children, suggesting that nutritional status may be closely related to peritonitis in dialysis patients (18). Serum albumin is also used as a leading indicator of nutritional status and morbidity/mortality in ESRD patients. A statistical analysis of the 1,723 paediatric cases registered in the US Renal Data System showed that the risk of mortality increases by 54% for every 1 g/dl decline in serum albumin at the start of dialysis (19). Previous Spanish study of paediatric PD showed that left ventricular diastolic dysfunction was an independent predictor of mortality. In addition, anaemia and hypoalbuminemia were risk factors for left ventricular diastolic dysfunction in these children (20). Therefore, children on PD should be closely monitored for nutritional status and serum albumin to improve clinical outcomes.

A previous study in the Netherlands reported 321 paediatric cases of PD-associated peritonitis. Of these, 9 had FP (2.9%), and 7/9 (78%) had received antibiotic therapy during the previous month. The history of antibiotics use was one of the most important risk factors for FP (2). The NAPRTCS data showed that 56% of patients who developed FP had exposure to antibiotics within 1 month prior to its development, with half of the antibiotic exposure due to treatment of bacterial peritonitis (1). In this study, the rate of antibiotics use during the month before the onset of peritonitis in the FP group (5/11) was higher than that in the BP group (18/113) (45.45 vs. 15.93%), while multivariate analysis did not identify antibiotics use during the month before onset of FP as an independent risk factor, which may be related to the small sample size in the FP group. Thus, FP still should be considered if patients have the history of recent antibiotics usage when there is no response to antibiotic therapy for peritonitis. In our centre, our population routinely receive fluconazole when treated for bacterial peritonitis. In this study, 5 patients received antibiotics within 1 month before the FP onset. Among these 5 patients, 4 patients who were transferred from another hospital (cases 6, 8, 10, 11) didn't receive fluconazole or nystatin when treated for bacterial peritonitis. The other one (case 9) received fluconazole when treated for bacterial peritonitis in our centre. The 2012 ISPD peritonitis prevention and treatment guidelines state that the use of oral nystatin or fluconazole be considered at the time of antibiotic administration to PD patients to reduce the risk of fungal peritonitis (7). Fungal prophylaxis with nystatin or fluconazole should as a part of routine prophylactic therapy.

Regarding the prognosis, the technical failure rate in the FP group was significantly higher than that in the BP group. All 11 FP cases (100%) were converted to HD, while only 6 BP patients (5.3%) were converted to HD. The study at a North America dialysis centre showed that approximately one-third of FP cases may resume PD (21). Previous study from Canada including 288 adult patients on PD showed that all 9 cases suffering from FP survived the episode, while only one patient resumed PD 12 months later (22). Moreover, another adult single-centre study from Korea indicated that among 94 episodes of FP, 59 patients (62.8%) required a change to haemodialysis and 27 patients (28.7%) died as a result of FP (23). In the future, the possibility of resuming PD after timely treatment will be investigated. In this study, hospital stays were longer (1.43 vs. 0.57 months) and healthcare expenses were higher (80,480.19 vs. 21,390.97 RMB) in the FP group than those in the BP group. In a North American multi-centre collaborative study, among 511 cases of peritonitis, 41 were FP (8.0%). The result showed younger age (<2 years) was the only risk factor identified, but FP was associated with a higher admission rate, a longer hospital stay, and a significantly higher catheter failure rate (6). High admission rates and high costs place tremendous financial burdens and mental stress on FP patients and their families.

## Conclusion

While the incidence of FP is low, its technical failure rate and its effects on peritoneal function, length of hospital stay, and healthcare expenses are significantly higher and longer than those of BP. It is critical to identify the risk factors for FP and detect and treat it in a timely manner. This study found that low serum albumin (≤30 g/L) was an independent risk factor for FP. Improving the positive rate of fungal culture and combining dialysate culture with the G test and GM test offers a meaningful method for improving the early diagnosis of FP. This study has some limitations. First, this was a retrospective analysis, which may lead to bias of data analysis. Second, this was a single-centre study with a small sample size, despite the use of similar treatment and management methods. Therefore, multi-centre prospective studies are needed to further evaluate the risk factors for PD-associated FP to better guide clinical treatment and improve prognosis.

## Data Availability Statement

The original contributions presented in the study are included in the article/supplementary material, further inquiries can be directed to the corresponding author/s.

## Ethics Statement

The studies involving human participants were reviewed and approved by Ethical Committee of Children's Hospital of Fudan University. Written informed consent to participate in this study was provided by the participants' legal guardian/next of kin.

## Author Contributions

XF, JCu, YZ, JiaoL, and JR contributed to the data analysis and drafted the manuscript. QS and HX contributed to the study design and critically revised the manuscript for important intellectual content. ZZ, JCh, JialL, and QM contributed to the patient follow-up and data collection. All authors have participated sufficiently in the work to take public responsibility for the content and approved the final version of the manuscript to be published.

## Conflict of Interest

The authors declare that the research was conducted in the absence of any commercial or financial relationships that could be construed as a potential conflict of interest.

## References

[1] Warady BA Bashir M Donaldson LA. Fungal peritonitis in children receiving peritoneal dialysis: a report of the NAPRTCS. Kidney Int. (2000) 58:384–9. 10.1046/j.1523-1755.2000.00176.x 10886585

[2] Raaijmakers R Schroder C Monnens L Cornelissen E Warris A. Fungal peritonitis in children on peritoneal dialysis. Pediatr Nephrol. (2007) 22:288–93. 10.1007/s00467-006-0289-x 17111161

[3] Goldie SJ Kiernan-Tridle L Torres C Gorban-Brennan N Dunne D Kliger AS. Fungal peritonitis in a large chronic peritoneal dialysis population: a report of 55 episodes. Am J Kidney Dis. (1996) 28:86–91. 10.1016/S0272-6386(96)90135-3 8712227

[4] Li PK Szeto CC Piraino B Bernardini J Figueiredo AE Gupta A. Peritoneal dialysis-related infections recommendations: 2010 update. Perit Dial Int. (2010) 30:393–423. 10.3747/pdi.2010.00049 20628102

[5] Miles R Hawley CM McDonald SP Brown FG Rosman JB Wiggins KJ. Predictors and outcomes of fungal peritonitis in peritoneal dialysis patients. Kidney Int. (2009) 76:622–8. 10.1038/ki.2009.202 19516241

[6] Munshi R Sethna CB Richardson T Rodean J Al-Akash S Gupta S. Fungal peritonitis in the standardizing care to improve outcomes in pediatric end stage renal disease (SCOPE) collaborative. Pediatr Nephrol. (2018) 33:873–80. 10.1007/s00467-017-3872-4 29313137

[7] Warady BA Bakkaloglu S Newland J Cantwell M Verrina E Neu A. Consensus guidelines for the prevention and treatment of catheter-related infections and peritonitis in pediatric patients receiving peritoneal dialysis: 2012 update. Perit Dial Int. (2012) 32 (Suppl. 2):S32–86. 10.3747/pdi.2011.00091 22851742 PMC3524923

[8] Shin JH Lee SK Suh SP Ryang DW Kim NH Rinaldi MG. Fatal Hormonema dematioides peritonitis in a patient on continuous ambulatory peritoneal dialysis: criteria for organism identification and review of other known fungal etiologic agents. J Clin Microbiol. (1998) 36:2157–63. 10.1128/JCM.36.7.2157-2163.1998 9650991 PMC105020

[9] Pimentel JD Mahadevan K Woodgyer A Sigler L Gibas C Harris OC. Peritonitis due to *Curvularia inaequalis* in an elderly patient undergoing peritoneal dialysis and a review of six cases of peritonitis associated with other Curvularia spp. J Clin Microbiol. (2005) 43:4288–92. 10.1128/JCM.43.8.4288-4292.2005 16082004 PMC1233934

[10] Miyazaki T Kohno S Mitsutake K Maesaki S Tanaka K Hara K. (1–>3)-beta-D-glucan in culture fluid of fungi activates factor G, a limulus coagulation factor. J Clin Lab Anal. (1995) 9: 334–9. 10.1002/jcla.1860090509 8531015

[11] Kocmanova I Racil Z Koukalova D Mayer J. 1,3-Beta-D-glucan measurement and its usefulness in the diagnosis of invasive fungal infections. Klin Mikrobiol Infekc Lek. (2008) 14:88–92. 18688768

[12] De Pauw B Walsh TJ Donnelly JP Stevens DA Edwards JE Calandra T. Revised definitions of invasive fungal disease from the European Organization for Research and Treatment of Cancer/Invasive Fungal Infections Cooperative Group and the National Institute of Allergy and Infectious Diseases Mycoses Study Group (EORTC/MSG) Consensus Group. Clin Infect Dis. (2008) 46:1813–21. 10.1086/588660 18462102 PMC2671227

[13] Karageorgopoulos DE Vouloumanou EK Ntziora F Michalopoulos A Rafailidis PI Falagas ME. beta-D-glucan assay for the diagnosis of invasive fungal infections: a meta-analysis. Clin Infect Dis. (2011) 52:750–70. 10.1093/cid/ciq206 21367728

[14] Douglas CM. Fungal beta (1,3)-D-glucan synthesis. Med Mycol. (2001) 39 (Suppl. 1):55–66. 10.1080/mmy.39.1.55.66 11800269

[15] Sulahian A Porcher R Bergeron A Touratier S Raffoux E Menotti J. Use and limits of (1-3)-beta-d-glucan assay (Fungitell), compared to galactomannan determination (Platelia Aspergillus), for diagnosis of invasive aspergillosis. J Clin Microbiol. (2014) 52:2328–33. 10.1128/JCM.03567-13 24740084 PMC4097729

[16] Pini P Bettua C Orsi CF Venturelli C Forghieri F Bigliardi S. Evaluation of serum (1 –> 3)-beta-D-glucan clinical performance: kinetic assessment, comparison with galactomannan and evaluation of confounding factors. Infection. (2016) 44:223–33. 10.1007/s15010-015-0849-8 26475482

[17] Oygar DD Altiparmak MR Murtezaoglu A Yalin AS Ataman R Serdengecti K. Fungal peritonitis in peritoneal dialysis: risk factors and prognosis. Ren Fail. (2009) 31:25–8. 10.1080/08860220802546446 19142806

[18] Chow KM Szeto CC Leung CB Kwan BC Law MC Li PK. A risk analysis of continuous ambulatory peritoneal dialysis-related peritonitis. Perit Dial Int. (2005) 25:374–9. 10.1177/089686080502500413 16022095

[19] Wong CS Hingorani S Gillen DL Sherrard DJ Watkins SL Brandt JR. Hypoalbuminemia and risk of death in pediatric patients with end-stage renal disease. Kidney Int. (2002) 61:630–7. 10.1046/j.1523-1755.2002.00169.x 11849406

[20] Garcia-Bello JA Ortiz-Flores J Torres DLRF Mendoza-Moreno GK Gomez-Tenorio C. Anemia and hypoalbuminemia as risk factors for left ventricular diastolic dysfunction in children with chronic kidney disease on peritoneal dialysis. Nefrologia. (2018) 38:414–9. 10.1016/j.nefroe.2018.06.010 30032857

[21] Nadeau-Fredette AC Bargman JM. Characteristics and outcomes of fungal peritonitis in a modern North American cohort. Perit Dial Int. (2015) 35:78–84. 10.3747/pdi.2013.00179 24497586 PMC4335931

[22] Levallois J Nadeau-Fredette AC Labbe AC Laverdiere M Ouimet D Vallee M. Ten-year experience with fungal peritonitis in peritoneal dialysis patients: antifungal susceptibility patterns in a North-American center. Int J Infect Dis. (2012) 16:e41–3. 10.1016/j.ijid.2011.09.016 22056278

[23] Chang TI Kim HW Park JT Lee DH Lee JH Yoo TH. Early catheter removal improves patient survival in peritoneal dialysis patients with fungal peritonitis: results of ninety-four episodes of fungal peritonitis at a single center. Perit Dial Int. (2011) 31:60–6. 10.3747/pdi.2009.00057 20505136

